# Bifidobacterium Longum: Protection against Inflammatory Bowel Disease

**DOI:** 10.1155/2021/8030297

**Published:** 2021-07-23

**Authors:** Shunyu Yao, Zixi Zhao, Weijun Wang, Xiaolu Liu

**Affiliations:** School of Chemistry and Biological Engineering, University of Science and Technology Beijing, Beijing 100083, China

## Abstract

The prevalence of inflammatory bowel disease (IBD), which includes ulcerative colitis (UC) and Crohn's disease (CD), increases gradually worldwide in the past decades. IBD is generally associated with the change of the immune system and gut microbiota, and the conventional treatments usually result in some side effects. Bifidobacterium longum, as colonizing bacteria in the intestine, has been demonstrated to be capable of relieving colitis in mice and can be employed as an alternative or auxiliary way for treating IBD. Here, the mechanisms of the Bifidobacterium longum in the treatment of IBD were summarized based on previous cell and animal studies and clinical trials testing bacterial therapies. This review will be served as a basis for future research on IBD treatment.

## 1. Introduction

Inflammatory bowel disease (IBD) is mainly manifested as chronic and recurrent inflammation in the gastrointestinal tract. It includes ulcerative colitis (UC) and Crohn's disease (CD) [1]. Although traditionally regarded as a disease prevalent in Western countries, the incidence of IBD is gradually increasing globally, especially in newly industrialized countries [2]. In the past decade, IBD has become a global public health challenge [3]. Its main symptoms include diarrhea, abdominal cramps, weight loss, fatigue, anemia, and extraintestinal symptoms (especially joint pain or arthritis). These will cause serious obstacles and troubles to human's normal life [4]. Most patients with IBD suffer from fecal incontinence and also face the risk of a weakened immune system and bowel cancer [5]. The occurrence of IBD is closely related to genetic susceptibility, environment, immune regulation dysfunction, gut microbiota, nutrition, and lifestyle [6]. However, the exact cause of IBD has yet to be determined, which makes it difficult to develop targeted treatments [4, 7].

At present, the commonly used drugs for the treatment of IBD include immunosuppressive drugs, biological agents, and antibiotics [8]. Among them, 5-aminosalicylic acid (5-ASA) is widely used in the treatment of IBD due to its good clinical efficacy [9]. However, taking this medicine will cause adverse reactions such as diarrhea, abdominal pain, headache, and nasopharyngitis, making the patient uncomfortable [8]. Monoclonal cytokines such as anti-TNF-*α* and IL-6 can also treat IBD, but the high production cost of this method makes it unacceptable for some patients [6]. Recently, studies have found that Bifidobacterium longum can be used as an adjuvant treatment for IBD [10]. Bifidobacterium longum belongs to the genera Actinomyces and Bifidobacterium. It is a gram-positive bacterium that performs anaerobic respiration [11]. The genus Bifidobacterium inhabits intestinal tracts of humans and animals. It is one of the first microorganisms to colonize the host gut [12]. It has more than 50 different species, of which, Bifidobacterium longum is one of the most abundant microorganisms in the intestines of infants and adults [8, 9]. It can be separated from a variety of animals, including intestines of babies and long-lived elderly [13]. Diseases inside and outside the intestine are closely related to the changes in the abundance of Bifidobacterium longum. Compared with healthy people, the abundance of Bifidobacterium longum flora in the stool of patients with intestinal diseases is much lower [14]. Bifidobacterium can be stably colonized in the human intestine. It has immune tolerance to the human body and will not cause rejection [15]. A large number of animal experiments and clinical studies have shown that Bifidobacterium longum can reduce the symptoms of colitis and relieve chronic inflammation [16]. However, the mechanisms of Bifidobacterium longum to treat IBD and regulate the intestinal immune system are still unclear. In this review, we will focus on the cell and animal experiments and clinical trials to summarize the mechanisms of Bifidobacterium longum on the prevention and treatment of IBD, which would provide a basis for subsequent therapeutic applications.

## 2. Interaction between Bifidobacterium longum and the Host

The human gastrointestinal environment can be regarded as a complex ecosystem. It contains trillions of microbes, which are usually called gut microbiota [17, 18]. Scientists have discovered that the composition of the gut microbiota and its metabolites plays an important role in protecting the intestinal barrier and regulating the immune balance [19]. Disturbances of the gut microbiota often occur in patients with intestinal diseases, such as irritable bowel syndrome, idiopathic chronic diarrhea, colorectal cancer, and IBD [20]. Some studies have shown that IBD usually causes general changes in the structure of the gut microbiota of patients, resulting in a decrease in the diversity and species abundance [21, 22]. The anaerobic species and short-chain fatty acid producers depleted, and the facultative anaerobic bacteria increased in the gut of patients [23]. Changes in gut microbiota will affect the normal operation of the mucosal immune system, leading to functional degradation [24]. Probiotics that promote the balance of gut microbiota play an important role in the treatment of IBD [25].

It is reported that the intervention of probiotics improved the gut microbiota and has an effective protective effect on the immune health of the host [26, 27]. The results of animal and clinical studies showed that products containing probiotics or prebiotics improved IBD by regulating proinflammatory signaling pathways and downregulating proinflammatory cytokines [7]. Bifidobacterium longum, as one of the most abundant members in the gut, can protect the intestinal epithelial barrier and tissue structure and balance the gut microbiota to alleviate the symptoms of colitis [28]. Moreover, Bifidobacterium can secrete a variety of active metabolites [29]. They influence the interaction between digestion, endocrine, cardiovascular, immune, and nervous systems to maintain the host in a healthy state [30, 31]. Bifidobacterium longum inhibits inflammation by regulating the balance of the immune system, improving the intestinal barrier function, and increasing acetate production [32]. This species has been widely used as a probiotic because of its beneficial effects on host health and has been recognized as safe by the United States Food and Drug Administration and the European Food Safety Authority [15].

## 3. Mechanisms of Bifidobacterium longum in Improvement of IBD

### 3.1. Bifidobacterium Longum and Antioxidant Activity

**Figure 1:**
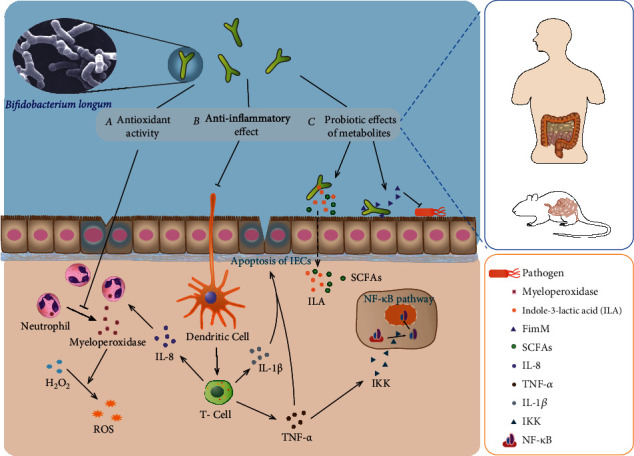
Protective mechanism of Bifidobacterium longum against intestinal inflammation.

Oxidative stress has been regarded as one of the major mechanisms involved in the pathophysiology of IBD [33]. It is characterized by the inability of the organism to detoxify reactive oxygen species (ROS) caused by a disequilibrium in the balance between their production and accumulation in cells and tissues [34]. The infiltration of immune cells occurred in active IBD as the prominent feature. More extensive recruitment of neutrophils and less of monocytes are the typical characteristics in lesion location. Myeloperoxidase (MPO), an abundant granule heme enzyme, is unique to both neutrophils and monocytes [35]. Through the halogenation or peroxidase cycle, MPO could generate reactive oxygen species (ROS) effectively [36]. ROS mainly includes the oxygen-containing ions, molecules, or groups with high activity. The abnormal accumulation of ROS will cause serious damage to normal physiological metabolic activity [37]. They induce fatty acid side-chain reactions to create lipid malondialdehyde and hydroperoxides, which results in the damage of biological macromolecules and causes the impairment of cell structure and function [37]. Substantial evidence shows that the imbalance between the accumulation of ROS and antioxidant activity is closely related to the incidence and severity of IBD. For IBD patients, oxidative stress occurs with the raise of ROS levels and decline of antioxidant levels, which leads to chronic tissue damage continuously [38, 39] (Figure 1).

Studies in cell and animal experiments have shown that Bifidobacterium longum strains regulate oxidative stress by enhancing the body's antioxidant activity and regulating the production and accumulation of ROS, thereby reducing the symptoms of IBD. B. longum 5(1A) administration in the dextran sulfate sodium- (DSS-) induced colitis in mice abated severe lesions in the colon with the decreased level of eosinophil peroxidase [40] (Figure 1). In addition, oral Bifidobacterium longum is also an effective treatment of ethanol-induced gastritis injury. Application of microbial inoculum downregulates the tumor necrosis factor (TNF) expression, myeloperoxidase activity, and hemorrhagic ulcerative lesions area [41]. Moreover, similar antioxidant effects have been found for the fermented products or metabolites of B. longum YS108R [24]. Without altering cell viability, B. longum CCFM752 supernatants increased intracellular antioxidative capacity with enhanced intracellular catalase activity and reduced NADPH oxidase activation [42].

Many anaerobic microorganisms remove ROS mainly by secreting and producing enzymes, such as NADH oxidase, NADH peroxidase, catalase and superoxide dismutase [43]. Currently, there are few studies concerning oxygen resistance and free radical scavenging genes or enzymes of B. longum, and there have been reports only about the strains NCC2705 [44], BBMN68 [43], and LTBL16 [45]. It has been found that B. longum LTBL16 had three peroxide oxidoreductase coding genes (LTBL16-000027, LTBL16-000028, LTBL16-000976) and one NADH oxidase coding gene (LTBL-001911), which can effectively remove ROS in bifidobacteria and improve oxygen resistance [45]. Recent studies have found that Bifidobacterium longum BBMN68 had an incomplete glutredoxin system. Thioredoxin and glutaredoxin make up the thioredoxin- and glutaredoxin-dependent reduction systems in Escherichia coli and many other bacteria and are responsible for maintaining a reduced environment in the cell cytosol [46]. Under oxidative stress, the genes *grxC1-* (BBMN68_125-) and *grxC2-* (BBMN68_1397-) encoding glutaredoxin, *trxB1-* (BBMN68_1345-) encoding thioredoxin reductase, and BBMN68_991-encoding thioredoxin are all upregulated [43]. Studies have found that when Bifidobacterium is under oxidative stress, thioredoxin reductase can respond positively to its transcription and translation [47]. In addition, the thioredoxin-dependent reduction system can reduce perredoxin and H2O2, scavenging free radicals, quenching singlet oxygen, and then maintaining the intracellular thioldisulfide balance [48]. Thus, the thioredoxin-dependent antioxidant system might be the major redox homeostasis system in strain BBMN68.

In mammals, several longevity proteins of the sirtuin family have been shown to play an antioxidant role by deacetylation activity. The cytosolic isoform SIRT2 is capable of deacetylating forkhead box protein FOXO1a and FOXO3a, thereby increasing FOXO-dependent transcription of antioxidant enzymes and reducing the cellular ROS level [49]. The probiotic (B. longum NCC2705) has the *Sir2* gene family and has antioxidant activity in the human body. BL-Sir2 regulated FOXO3a mediated antioxidant genes, deacetylated *σ*H, and increased the activity of manganese superoxide dismutase and catalase and reduced ROS [44]. In addition, a *Sir2*-encoding gene (LTBL16-002010) was also found in B. longum LTBL16, which could improve FOXO-dependent transcription of antioxidant enzymes encoding genes and reduce ROS levels in cells [45]. Therefore, Bifidobacterium longum can suppress oxidative stress and stimulate the production of antioxidants, thereby reducing the oxidative damage of intestinal tract of IBD (Figure 1).

Bifidobacterium longum can protect intestinal epithelial cells by different mechanisms. These include (a) Bifidobacterium longum can decline myeloperothe xidase activity and the production of ROS, suppress oxidative stress, and reduce the damage of the tintestinal tract. (b) Bifidobacterium longum can downregulate inflammatory cytokines and inhibit NF-*κ*B pathway to regulate the intestinal immune system and protect intestinal epithelial cells. (c) Bifidobacterium longum can produce various metabolites to enhance adhesion to the intestinal tract and inhibit harmful bacteria. It can also participate in immune regulation. Bifidobacterium longum was photographed by Mark Schell, University of Georgia, Athens, GA [50].

### 3.2. Bifidobacterium longum Reduces the Inflammatory Cytokine Expression in the Intestine

In vitro experiments and animal models indicate that Bifidobacterium longum has anti-inflammatory effects on intestinal diseases (Table 1 and Table 2). Bifidobacterium longum can reduce spontaneous and chemically induced colitis by regulating cytokines or inducing immune regulation mechanisms in a specific way [51]. The intestine is an important immune organ. Goblet cells in the intestine produce mucus to fight off invading pathogens. Under the mucus, intestinal epithelial cells and various immune cells form another defense barrier to prevent the invasion of pathogenic microorganisms [52]. These cells can specifically secrete various cytokines to regulate the immune system. For example, Th1 cells can secrete tumor necrosis factor *α* (TNF-*α*) to initiate a variety of proinflammatory responses [53]. Th17 cells are involved in the activation and recruitment of neutrophils [54]. Treg cells can express the transcription factor forkhead box P3 (FOXP3) and secrete the anti-inflammatory cytokine IL-10, thereby inhibiting a strong inflammatory response [55].

Under normal circumstances, the mucosal cells of the intestine can keep the proinflammatory and anti-inflammatory cytokines in a relatively balanced state [51]. In the intestines of patients with IBD, this balance is disrupted. The increase in the number and activity of proinflammatory cytokines in the mucosa leads to damage and inflammation of the intestinal tissues [56]. In the process of IBD, immune cells are activated after receiving a stimulating signal. A large number of inflammatory cytokines are secreted, including tumor necrosis factor (TNF-*α*), interleukin (IL-1*β*), IL-6, and ROS [57] (Figure 1). An increase in intestinal epithelial cell (IEC) apoptosis is a major characteristic of IBD. Studies have shown that excessive TNF-*α* can destroy the integrity of the intestinal epithelium and induce apoptosis of IECs [58] (Figure 1). The study of T cell metastasis showed that the content of TNF-*α* in the intestinal tract of colitis increased significantly [59]. In the study of various strains of Bifidobacterium longum, it was found that after incubating cells with probiotics, the level of TNF-*α* was significantly reduced. The disease can be alleviated by the neutralizing effect of TNF-*α* [51, 60, 61].

Furthermore, TNF-*α* induces inflammatory responses with the expression of proinflammatory cytokines, including IL-1*β*, IL-6, and IL-8 [62]. The IL-1*β* is produced by IECs in a paracrine manner. It could disrupt the maturation and function of IECs resulting in exerting major epithelial barrier alterations [63]. As a pleiotropic cytokine, IL-6 plays a central role in immunoregulation, inflammation response, and oncogenesis. Anti-IL-6 monoclonal antibody effectively suppresses chronic intestinal inflammation in mouse models [64]. A previous research demonstrates that proinflammatory molecules like IL-8 could be induced by enteropathogenic bacteria colonizing in the gut. As a consequence, neutrophils and other inflammatory cells will be recruited [65]. Infiltration of neutrophils may perpetuate inflammation and result in cell damage, epithelial barrier dysfunction, and diarrhea [66]. Marzia et al. used B. longum and macrophages to conduct a simulation study of the intestinal epithelial barrier function. It was found that IL-10 was induced by probiotics significantly. On the contrary, the production of IL-1*β* and IL-6 was downregulated by 70% and 80%, respectively [67]. Similarly, after coincubation with B. longum HT-CECT-7347, HT29 cells stimulated by TNF-*α* displayed a drastic dose-dependent decline in IL-8 production [62]. In addition, it was found that after treatment with Bifidobacterium longum, colitis mice alleviated inflammation, and the content of short-chain fatty acids in the intestinal tract also increased. The regulation of immunity by short-chain fatty acids (SCFAs) is mainly mediated by activation of free fatty acid receptor 2 (FFA2) or inhibition of histone deacetylase (HDAC) [68]. As the main receptor of SCFA, FFA2 is expressed on immune cells and inhibits the NF-*κ*B signaling pathway to produce anti-inflammatory effects [69]. HDACs are generally expressed in immune, endothelial, and vascular smooth muscle cells [70]. Inhibition of HDAC activity causes an open structure of DNA/chromatin, which facilitates the regulation of the expression of transcription factors, such as NF-*κ*B and FOXP3 [68]. Therefore, B. longum can regulate intracellular signaling pathways and decrease the level of IL-1*β*, IL-6, and IL-8, reduce the alterations of the in vitro epithelial barrier induced by DSS, and regulate the inflammatory response [59, 71, 72] (Figure 1).

NF-*κ*B plays a crucial role in a variety of immune and inflammatory reactions in the intestine. It can participate in the induction and regulation of the related gene expression [75]. Studies have found that TNF-*α* acts through the activation of TNF receptors. This activation triggers a series of intracellular events that result in the activation of the transcription factor NF-*κ*B [76]. Its activation level is closely related to the severity of intestinal inflammation. Upon receipt of a proinflammatory stimulus, IKK phosphorylates inhibitory kB (IkB) molecules, releases NF-*κ*Bp50-p65 heterodimeric protein, migrates to the cell nucleus, and binds to specific kB sites (Figure 1). Genes encoding cytokines and chemokines, cell adhesion molecules, and immune receptors will be activated and transcribed to produce important mediators of inflammation [77, 78]. In an ethanol-induced gastroenteritis study, the oral administration of B. longum LC67 in mice was found to suppress the TNF-*α* expression and NF-*κ*B activation in mucosal cells, restore the gut microbiota disturbance, and alleviate ethanol-induced GI inflammation [28]. For the mice with high-fat diet- (HFD-) induced obesity, B. longum alleviated colitis by regulating NF-*κ*B activation through the inhibition of the production of harmful substances in the gut microbiota [74]. Further research showed that Bifidobacterium longum could prevent the nuclear localization of NF-*κ*B-p65 in the damaged intestine to a certain extent and increase the expression of NF-*κ*B-p65 in the cytoplasm [62, 79].

Besides, probiotics can secrete tryptophan metabolites to maintain the healthy homeostasis of the host [80]. It has previously been reported that a number of colonizing intestinal bacteria, particularly Gram-negative organisms, can metabolize the amino acid tryptophan to improve health and provide immune protection [81]. Bifidobacterium longum subsp. infantis can produce indole-3-lactic acid (ILA) in its culture medium as an anti-inflammatory molecule (Figure 1). This molecule reduces the IL-8 response after IL-1*β* stimulus. It interacts with the transcription factor aryl hydrocarbon receptor (AHR) and prevents transcription of the inflammatory cytokine IL-8 [82]. In addition, it could significantly attenuate lipopolysaccharide- (LPS-) induced activation of NF-*κ*B in macrophages and significantly attenuate TNF-alpha and IL-8 in intestinal epithelial cells to protects gut epithelial cells [82, 83]. ILA increased the mRNA expression of the aryl hydrogen receptor- (AhR-) target gene *CYP1A1* and nuclear factor erythroid 2-related factor 2- (Nrf2-) targeted genes glutathione reductase 2 (*GPX2*), superoxide dismutase 2 (*SOD2*), and NADPH dehydrogenase (*NQO1*) and protects gut epithelial cells in culture via activation of the AhR and Nrf2 pathway [83]. Therefore, Bifidobacterium longum can reduce the production of proinflammatory cytokines, inhibit the activation of NF-*κ*B induced by TNF-*α*, and improve the symptoms of IBD (Figure 1).

### 3.3. Bifidobacterium longum Enhances the Intestinal Barrier Function

Intact intestinal epithelial cells can ensure the normal intestinal function. It can resist pathogenic microorganisms and harmful substances in the intestinal environment to avoid damage [85]. Good intestinal barrier function requires tight junctions between intestinal epithelial cells [86]. In IBD, the intestinal permeability of the patient's intestinal mucosa increases, and the expression of the tight junction protein (TJP) decreases, which affects the protective function of the intestine and causes inflammation [87]. Inflammation of the intestinal epithelial mucosa will exacerbate this phenomenon, leading to a further decrease in TJP and forming a vicious circle [88]. Studies have shown that feeding mice with B. longum YS108R can improve the mucosal barrier damage induced by DSS and increase the expression of TJP and mucin2 to alleviate colitis [24]. In a similar experiment on 2,4,6-trinitrobenzenesulfonic acid- (TNBS-) induced colitis mice, it was found that the expression of tight junction proteins ZO-1, occluding, and claudin-1 in the colon was significantly reduced, but this phenomenon was alleviated after feeding with B. longum HB5502 [84].

Moreover, some studies have shown that IBD patients were accompanied with weight loss, inflammatory cell infiltration, anemia, decrease of colon length, and damage of the mucosal layer [89]. Mice with colitis induced by chemical reagents are often used as models to obtain symptoms similar to IBD for research on the treatment of related diseases [90]. Feeding mice with Bifidobacterium longum strains Bif10 and Bif16 could reduce their crypt deformation, diarrhea, etc. The decrease in colon length was alleviated, and the survival rate was improved [59]. Compared to the control group, the infiltration of inflammatory cells in the colon tissue of the B. longum ATCC 1570 treatment group was improved. Crypt alterations and ulceration areas were not observed in the epithelium [71]. Similarly, Bifidobacterium longum LC67 can alleviate TNBS-induced colon shortening in mice. Myeloperoxidase activity is also reduced. At the same time, the edema and destruction of colonic epithelial cells have been relieved, and the expression of the colonic tight junction protein has been restored [79]. In addition, the study found that after treatment with Bifidobacterium longum, the content of SCFAs in the intestinal tract of colitis mice also increased. SCFAs, as metabolites of the gut bacteria, are used by epithelial cells as their primary energy source to promote the health of the GI system [91]. SCFAs improves the expression of connexin in intestinal epithelial cells by enhancing the expression of the *MUC2* gene and activating the AMP-activated protein kinase (AMPK) pathway [92]. Moreover, SCFA has an impact on the population and function of innate immune cells through G-protein coupled receptor signaling and HDAC inhibition and plays an important role in maintaining the intestinal barrier function [91, 93].

### 3.4. Bifidobacterium longum Regulates Gut Microbiota

In normal individuals, symbiosis exists between the gut microbiota and the host. This harmonious and stable symbiotic relationship can regulate mucosal immunity and prevent the colonization of pathogens in the intestine [94] (Figure 1). Recently, studies have revealed that gut microbiota imbalance played a vital role in the causation of various diseases including IBD [95]. The improvement of gut microbiota composition has been proposed as an effective auxiliary method for the treatment of certain intestinal inflammatory diseases [96]. A previous research has revealed that the gut flora of DSS-treated mice changed significantly in comparison with the control group. The abundance of gut microbiota was reduced, and the bifidobacteria supplementation alleviated the changes of gut microbiota induced by DSS [72]. B. longum YS108R can produce abundant extracellular polymeric substances (EPS). After feeding the fermented milk to DDS-induced colitis mice, it was found that the gut microbiota was adjusted, and pathogenic bacteria such as Enterobacteriaceae were also suppressed [24].

Probiotics in the intestine can release many biologically active peptides, bringing countless benefits to the health of the host [97]. Adhesion to the gastrointestinal tract is considered to be important for bifidobacteria to colonize the human gut and exert their probiotic effects. FimM is a novel surface adhesin that is mainly present in B. longum strains. Under normal circumstances, FimM may block pathogen access to the mucus layer by binding to mucins. Under pathogen invasion, FimM could competitively inhibit pathogen adhesion by binding to fibronectin and fibrinogen [98]. In addition, Bifidobacterium supplementation increased the level of intestinal SCFAs and inhibited the abundances of pathobionts at the genus level. Bacterial components of B. longum fed mice were slightly different from those of healthy mice [59]. As the final products of anaerobic intestinal microbiota fermentation, SCFAs have beneficial effects in accelerating intestinal movement and modulating the body immune system. They can also increase the risk of metabolic syndrome and reduce plasma cholesterol levels [99, 100] (Figure 1). Another study stated that B. longum KACC 91563 exists favorable impacts on increase of the SCFA content in feces of normal dogs and improves the gut microbiota structure [101]. The study found that B. longum BB536 had a synergistic effect with gut microbiota, which is helpful to maintain body homeostasis, and reduce the probability of gastrointestinal and allergic diseases [18]. These results indicated that Bifidobacterium longum had active influences on host healthy through restoring the gut microbiota balance.

## 4. Application of Bifidobacterium longum in Clinical Trials

Many clinical trials have shown that using Bifidobacterium longum can effectively improve the symptoms of IBD (Table 3). In comparison with placebo-treated subjects, B. longum 536 can improve the clinical symptoms of patients with mild to moderately active UC. 8 weeks after treatment, disease activity and clinical scores are greatly reduced [102]. 12 weeks after treatment with Bifidobacterium longum in patients with IBS-D, proinflammatory cytokines (IL-6, IL-8, and tumor necrosis factor TNF-*α*) were decreased, and intestinal permeability and gastrointestinal symptoms were improved [103]. Besides, Bifidobacterium longum is also used together with other ingredients to achieve better results. For example, when it was used together with the prebiotic synergy 1, the CD activity and histological score were reduced [104]. Bifidobacterium longum and inulin-oligofructose were provided to UC patients. 4 weeks after treatment, it was found that the expression of *β*-defensin, IL-1*α*, and TNF-*α* genes was decreased. At the same time, rectal biopsy was improved, inflammation was reduced, and epithelial tissue was regenerated [105].

When the probiotic product VSL#3 embodying Bifidobacterium longum was administered to patients with active UC, the symptoms of enteritis were effectively relieved 6 weeks after treatment, and there was no adverse reactions [79]. In 2009, similar results were obtained in experiments on patients with mild to moderate UC, and the disease activity index was also reduced [106]. Fedorak et al. assessed the preventive effect of VSL#3 against postoperative CD recurrence and found that the reduction of the proinflammatory cytokines in the patient's intestinal mucosa was owed to VSL#3, and the postoperative recurrence rate was also remained at a low level [107]. Similarly, the use of VSL#3 was found to be able to alleviate the pain of IBD patients to varying degrees and has great potential for disease treatment [108–111]. Therefore, the results of clinical trials prove that the use of Bifidobacterium longum alone or in combination with other probiotics can effectively improve the symptoms of IBD patients. Bifidobacterium longum can be used as an effective preventive or auxiliary treatment for IBD.

## 5. Future Perspectives of Bifidobacterium longum-Associated Therapy in IBD

In the past decades, the use of genetic engineering and biological engineering to express proteins or polypeptides with specific functions using bifidobacteria as vectors has become a new therapeutic method [113, 114]. Bifidobacterium is an excellent candidate for the development of living vectors for the production and delivery of heterologous proteins on mucosal surfaces. Bifidobacterium longum, which is a probiotic, can be colonized in the intestine for a long time, and it is immune tolerant to the human body. In the case of long-term use, it will not cause rejection by the human body [115]. However, compared to the use of a single bacterial agent, the effect of using a composite bacterial agent is more significant. The optimal dose and treatment time of the bacterial agent in the course of use and its molecular mechanism of action have not yet been determined. In addition, probiotic preparations take a long time to be effective [116]. In the treatment of severe acute inflammatory bowel disease, chemical drugs and surgical treatment are still the first choice [117]. Therefore, the above issues will be the focus of future research.

## 6. Conclusions

Bifidobacterium longum is a symbiotic bacterium existed in the human gastrointestinal tract. Both animal and clinical trials have found and demonstrated that Bifidobacterium longum had preventive and protective impacts on IBD. Bifidobacterium longum can change the structure of the gut microbiota, induce and regulate immune responses, and reduce the expression of inflammatory cytokines and ROS in the intestine. Besides, it can also maintain the normal intestinal barrier function by increasing the expression of the TJP protein. Therefore, Bifidobacterium longum has great potential and can be used as a prevention, replacement, or adjuvant treatment for IBD.

## Figures and Tables

**Table 1:** Effects of B. longum strains in modulating inflammation based on in vitro and ex vivo studies.

Strains	Dose	Cell	Effect	Ref.
B. longum CECT-7347	2 × 10^9^ cells/mL	HT-29 cell	IL-8 ↓	[62]
B. longum Bif10 and Bif16	1 × 10^10^ CFU/mL	RAW264.7 cell	TNF-*α*, IL-1*β*, IL-6 ↓ SCFA ↑	[59]
B. longum BB536	5 × 10^8^ cells/mL	PIE cell	TNF-*α*↓	[60]
B. longum KACC 91563	1 × 10^6^, 10^7^, 10^8^ CFU/well	Splenocytes macrophages	TNF-*α* ↓, IgE ↓ IL-2, 4, 6, 10, IFN-*γ* ↓	[51]
B. longum R0033	100 : 1 for bacteria to cell ratio	HT-29 cell	TNF-*α*, IL-8 ↓	[61]
B. longum 5^1A^	1 × 10^3^, 10^5^ CFU/well	Keratinocyte fibroblast cell	IL-6, IL-8 ↓	[73]
B. longum BL05	5 × 10^4^, 10^5^, 10^6^ CFU/well	HT-29 cell THP-1 cell	IL-10 ↑ IL-1*β*, IL-6 ↓	[67]
B. longum LC67	1 × 10^3^, 10^5^ CFU/mL	KATO III cells	NF-*κ*B ↓ IL-8 ↓	[41]
B. longum LC67	1 × 10^4^, 10^6^ CFU/mL	Caco-2 cells	NF-*κ*B ↓	[28, 74]

**Table 2:** Animal studies of B. longum strain effects in modulating inflammation.

Strains	Dose	Model	Effect	Ref.
B. longum Bif10 and Bif16	5 × 10^9^ CFU/mouse/day	DSS-induced colitis in mice	SCFA ↑ TNF-*α*, IL-1*β*, IL-6 ↓	[59]
B. longum 5 (1A)	1 × 10^8^ CFU/mouse/day	DSS-induced colitis in mice	IL-1 ↓ MPO ↓	[40]
B. longum YS108R	1 × 10^9^ CFU/mouse/day	DSS-induced colitis in mice	IL-10 ↑ TNF-*α*, MPO, IL-1*β*, IL-6, IL-17A ↓	[24, 72]
B. longum ATCC 15707	1 × 10^7^ CFU/kg/day	DSS-induced colitis in mice	SCFA ↑ TNF-*α*, IL-6, TGF-*β* ↓	[71]
B. longum HB5502	4 × 10^9^ CFU/day	TNBS-induced colitis in mice	HMGB1 ↓	[84]
B. Longum LC67	1 × 10^9^ CFU/mouse/day	Ethanol-induced gastritis in mice	NF-*κ*B, CXCL4, TNF ↓	[41]
B. longum LC67	1 × 10^9^ CFU/mouse/day	High-fat diet-induced colitis in mice	AMPK ↑ NF-*κ*B ↓	[74]
B. longum LC67	1 × 10^9^ CFU/mouse/day	TNBS-induced colitis in mice	NF-*κ*B, MPO ↓	[79]

**Table 3:** Clinical evidence for B. longum with IBD.

Strains	Number of patients (age)	Length of treatment	Dose	Effect	Ref.
B. longum 536	56 (31-58 years old)	8 weeks	2 − 3 × 10^11^ CFU/day	Clinical remission UC disease activity index ↓	[102]
B. longum ES1	16 (16-65 years old)	12 weeks	1 × 10^9^ CFU/day	Proinflammatory cytokines ↓ Improved intestinal permeability	[103]
B. longum and synergy 1	35 (18-79 years old)	6 months	4 × 10^11^ CFU/day	CD activity ↓ TNF-*α* ↓	[104]
B. longum and inuli	18 (24-67 years old)	4 weeks	4 × 10^11^ CFU/day	Inflammatory parameters ↓	[105]
VSL #3	147 (26-52 years old)	12 weeks	7.2 × 10^12^ CFU/day	Induction of remission in mild-to-moderate UC	[106]
VSL #3	119 (25-49 years old)	9 months	1.8 × 10^10^ CFU/day	Inflammatory cytokine levels ↓	[107]
VSL #3	131 (33-62 years old)	8 weeks	3.6 × 10^9^ CFU/day	Clinical scores in UC ↓	[109]
B. longum 536	12 (28-45 years old)	1 months	4 × 10^9^ CFU/day	Improvement of gut microbiota	[112]
